# COX-2–PGE_2_ Signaling Impairs Intestinal Epithelial Regeneration and Associates with TNF Inhibitor Responsiveness in Ulcerative Colitis

**DOI:** 10.1016/j.ebiom.2018.08.040

**Published:** 2018-09-03

**Authors:** Yuan Li, Christoffer Soendergaard, Fredrik Holmberg Bergenheim, David M. Aronoff, Ginger Milne, Lene Buhl Riis, Jakob Benedict Seidelin, Kim B. Jensen, Ole Haagen Nielsen

**Affiliations:** a Department of Gastroenterology, Herlev Hospital, University of Copenhagen, Herlev DK-2730, Denmark; b Division of Infectious Diseases, Department of Medicine, Vanderbilt University Medical Center, Nashville, TN 37232, USA; c Division of Clinical Pharmacology, Department of Medicine, Vanderbilt University Medical Center, Nashville, TN 37232, USA; d Department of Pathology, Herlev Hospital, University of Copenhagen, Herlev DK-2730, Denmark; e BRIC - Biotech Research and Innovation Centre, University of Copenhagen, Ole Maaløes Vej 5, Copenhagen DK-2200, Denmark; f Novo Nordisk Foundation Center for Stem Cell Biology, Faculty of Health Sciences, University of Copenhagen, Copenhagen DK-2200, Denmark

**Keywords:** COX-2, Intestinal epithelial cells, Monocytes, Prostaglandin E_2_, Ulcerative colitis

## Abstract

**Background:**

Inhibition of tumor necrosis factor-α (TNF) signaling is beneficial in the management of ulcerative colitis (UC), but up to one-third of patients do not have a clinical response of relevance to TNF inhibitors during induction therapy (i.e. primary non-responders [PNRs]). Through production of prostaglandins (PGs) and thromboxanes, cyclooxygenase-2 (COX-2) affects inflammation and epithelial regeneration and may in this way be implicated in treatment resistance to TNF inhibitors.

**Methods:**

In this study, COX-2 expression was analyzed in human intestinal biopsies and patient-derived monocytes, and the downstream consequences of COX-2 activity was evaluated by assessing the influence of the down-stream effector, PGE_2_, on intestinal epithelial stem cell self-renewal and differentiation using primary human intestinal organoids (“mini-guts”).

**Findings:**

We found that TNF stimulation induced *COX-2* expression in monocytes isolated from responders (Rs), whereas *COX-2* expression was constitutively high and non-inducible in monocytes from PNRs. Additionally, PGE_2_ in combination with proliferative signals transformed human intestinal epithelial cells to a proinflammatory state akin to flaring UC, whereas PGE_2_ in combination with differentiation signals supported robust mucin induction.

**Interpretation:**

Our work indicates that COX-2-PGE_2_ signaling could be a novel target for the management of PNRs to TNF inhibitors. We additionally demonstrate that COX-2–PGE_2_ signaling has dual functions during tissue repair and normal lineage differentiation, explaining in part the lack of response to TNF inhibitors among PNRs.

**Fund:**

This work was funded by grants from the Novo Nordisk Foundation, the Lundbeck Foundation, the Vanderbilt Digestive Disease Research Center, NIH Grants, Aase and Ejnar Danielsen's Foundation and the A.P. Møller Foundation.

## Highlights

• Monocytes derived from primary non-responders to therapy with TNF inhibitors have a higher basal expression level of *COX-2.*
• The sustained induction of PGE_2_ maintains an inflammatory state of TNF-stimulated intestinal epithelium.
• The COX-2–PGE_2_ pathway might be a druggable pathway to affect TNF inhibitor responsiveness.

**Research in Context**

**Evidence before this Study**

The COX-2-PGE2 pathway has been suggested to transiently promote intestinal repair in murine studies. Moreover, the use of COX-2 inhibitors in patients with ulcerative colitis (UC) has been linked to an increased risk of flaring disease, although these observations have recently been questioned. Expression analysis using material from patients suffering from UC stratified based on their response to TNF inhibitors, suggests that COX-2 levels are elevated in the group of non-responders prior to TNF inhibitor therapy.

**Added Value of this Study**

Our aim was to address whether changes in the COX-2–PGE_2_ pathway could explain the downstream consequences of aberrant COX-2 expression and the associated link to a relevant response to TNF inhibitor therapy using patient derived material. We demonstrate for the first time that monocytes from patients not responding to TNF inhibitors have higher basal levels of *COX-2* as compared to monocytes isolated from responders. Additionally, PGE_2_, which is the major downstream effector of COX-2, exacerbates the expression of proinflammatory cytokines upon TNF stimulation in human intestinal epithelial cells, thereby ameliorating the inflammatory response, and potentially it impairs the reestablishment of a functional epithelial barrier.

**Implications of all the Available Evidence**

The present work indicates that the COX-2-PGE_2_ pathway should be explored as a target for primary non-responders to TNF inhibitor therapy as well as a prognostic biomarker for the TNF inhibitor responsiveness.

Alt-text: Unlabelled Box

## 1 Introduction

Ulcerative colitis (UC) and Crohn's disease (CD) are the two main subtypes of inflammatory bowel disease (IBD), both with increasing incidence and prevalence worldwide [1]. UC is a chronic disease of unknown etiology characterized by chronic inflammation of the colon and rectum with a progressive and remitting/relapsing course [2]. Tumor necrosis factor-α (TNF) is one of the most important mediators of the proinflammatory response in UC. Over the past two decades, biologics acting by inhibiting TNF through genetically engineered monoclonal antibody constructs (TNF inhibitors) have revolutionized the management of UC [3]. However, up to one-third of patients fail to achieve any clinical response of relevance within the induction phase (i.e., 14 weeks after initiation of treatment) and are referred to as *primary non-responders* (PNRs) [3,4]. It is crucial to identify the mechanisms governing the response to TNF inhibitors because this may allow for early identification of PNRs and optimization of treatment strategies, as well as avoidance of superfluous treatment costs.

In addition to elevated levels of TNF in the inflamed colon, UC is accompanied by colonic epithelial barrier defects [5]. Ample evidence supports that loss of epithelial integrity contributes to prolonged mucosal inflammation in UC, and that epithelial regeneration is crucial for the induction of mucosal healing [2,6,7]. Due to their capability of self-renewal and differentiation, intestinal epithelial stem cells located at the base of intestinal crypts play a decisive role in the epithelial regeneration process [8]. Upon damage monocytes/macrophages are recruited to the sites of injury where they constitute a major source of TNF [9,10]. Differences have been observed in monocytes derived from responders (Rs) when compared to PNRs with UC [11]. It is consequently of interest to investigate whether these differences extend into the TNF-induced inflammatory response thereby altering responsiveness to TNF inhibitors and affecting epithelial regeneration.

The cyclooxygenase (COX) enzymes consist of two isoforms, COX-1 and COX-2, which can metabolize released arachidonic acid from cell membranes via the common precursor prostaglandin H_2_ (PGH_2_) into different prostanoids, comprising PGE_2_, PGD_2_, PGF_2α_, PGI_2_, and thromboxane A_2_ (TxA_2_) [12,13]. Unlike COX-1, which is constitutively expressed in multiple tissues, including the gastrointestinal tract, the expression of COX-2 is typically induced by inflammation, such as TNF stimulation [14,15]. Additionally, COX-2 inhibitor treatment of UC patients has for a long time been linked with an increased risk of flaring disease [16,17], although a recently published meta-analysis has questioned this generalization in patients with IBD [18]. Further, the most abundant COX-derived metabolite [15], PGE_2_, has been shown to be required for intestinal wound repair by promoting the differentiation of wound-associated epithelial cells (WAE) [19]. Moreover, inhibition of the COX-2–PGE_2_ pathway in myofibroblasts has been shown to increase the susceptibility to dextran sodium sulfate (DSS)–induced experimental colitis in mice [20]. These facts combined indicate that regulation of the COX-2–PGE_2_ pathway might be involved in epithelial regeneration and could affect the clinical response of TNF inhibitor treatment in UC. Therefore, based on the current knowledge of involvement of eicosanoids in a wide range of physiological and pathological processes in the gastrointestinal tract [21], we hypothesized that responsiveness to TNF inhibitors might be influenced by an altered regulation of prostanoid synthesis in the intestinal tract of patients with UC. Using patient-derived material, we determined the TNF responsiveness of monocytes derived from TNF inhibitor treatment responders (Rs) or primary non-responders (PNRs), with emphasis on the COX-2 signaling pathway. We meticulously mapped the effects in vitro on cultures of intestinal epithelial stem cells with the aim to elucidate how the COX-2–PGE_2_ pathway affects the responsiveness to anti-TNF therapy in patients with UC.

## 2 Materials and Methods

### 2.1 Study Population

Patients aged 18 to 75 years were recruited at the East Danish IBD Centre at Herlev Hospital, University of Copenhagen, Denmark. Following informed consent, all eligible patients had their diagnosis of UC verified by well-established criteria [22], and their disease activities were graded at the time of enrollment in accordance with the Mayo score (a combined score consisting of three clinical items: (stool frequency [0–3], rectal bleeding [0–3] and physicians' global rating [0–3]) as well as an endoscopic score [0–3]). Thus, the higher the total Mayo score, the more severe the UC: a score of 0–2: quiescent UC; 3–5: mild UC; 6–9: moderate UC; and 10–12: severe UC [23,24]. All patients in this study were initially treated with the TNF inhibitor, infliximab used as 1^st^ choice at the East Danish IBD Centre administered intravenously in the dosage of 5 mg/kg bodyweight at week 0, 2 and 6 (induction regimen) and then every 8 weeks (the latter maintenance regimen for Rs only). Thus, at week 14 all patients were assessed as Rs or PNRs to TNF inhibitor treatment, and a R was defined as a patient with a decrease in Mayo score of three points or more during the initial 14 weeks of treatment [25]. In turn, a PNR was defined as a patient without clinically relevant improvement in Mayo score (i.e., a decline in score from baseline of <3 despite induction therapy with a TNF inhibitor). Except two PNRs who underwent colectomy, all the PNRs were switched out of therapeutic class to e.g., the anti-integrin, vedolizumab, combined with other treatments (e.g., thiopurines like azathioprine, glucocorticoids, and 5-aminosalicylic acid [5-ASA] at week 14). When blood samples were obtained, the Rs were placed on maintenance TNF inhibitor therapy while PNRs received second-line therapy (Supplemental Table S1), and the median clinical Mayo score in both Rs and PNRs was 0 (Supplemental Table S1). Blood samples were obtained from patients with UC, including Rs (n = 10) and PNRs (n = 10), and healthy control subjects (n = 6). Colonic biopsies for COX-2 immunohistochemistry (IHC) were collected from routine colonoscopies of patients with UC, including Rs (n = 10) and PNRs (n = 10), and healthy control subjects (n = 10). Control samples were attained from patients who underwent such an examination but in whom all clinical investigations subsequently turned out to be normal, e.g., a diagnosis of irritable bowel syndrome was reached [26]. Information on patients included is provided in Supplemental Table S1.

### 2.2 Monocyte Isolation and Stimulation

Blood was drawn from TNF inhibitor–experienced patients with UC and diluted 1:1 with phosphate-buffered saline (PBS), following isolation of peripheral blood mononuclear cells (PBMCs) by Ficoll-Paque density gradient centrifugation (GE Healthcare, Uppsala, Sweden). PBMCs were used for monocyte isolation of CD14^+^ monocytes by negative immunomagnetic bead separation using the Monocyte Isolation Kit II (Miltenyi Biotec, Auburn, CA, USA). After isolation, 1 × 10^6^ monocytes per well were plated in 24-well plates (TPP, Trasadingen, Switzerland). Then 1 ml of growth medium (RPMI-1640 medium containing 10% human serum, 50 IU/ml penicillin, 50 μg/ml streptomycin, and 0.5 mg/ml gentamycin) was added to each well, and the plates were incubated at 37 °C with 5% CO_2_ pressure. Cells were cultured overnight and were subsequently stimulated with 10 ng/ml of recombinant TNF (cat. no. 210-TA, R&D Systems, Minneapolis, MN, USA) for 8 h. Unstimulated cells were used as controls.

### 2.3 Crypt Isolation and Organoid Culture

Colonic biopsies were isolated from the sigmoid part of the colon of control patients and washed thoroughly in cold Dulbecco's phosphate-buffered saline (DPBS), and epithelial cells were isolated after chelation using EDTA (8 mM) for 30 min on ice, followed by vigorous shaking. After centrifuging at 125 ×*g* at 4 °C for 5 min, the supernatant was removed, and 1 ml of Ad-Df^+++^ (Advanced DMEM-F12 plus Glutamax, HEPES, and penicillin/streptomycin [all obtained from Invitrogen, Carlsbad, CA, USA]) was added to resuspend the pellet. The number of crypts was counted under a microscope. For one well on a 48-well plate, approximately 100 crypts were suspended in 25 μl of a mixture (1:1) of Matrigel (Corning, New York, NY, USA) and Ad-Df^+++^. The plate was then placed in a 37 °C incubator for 15 min to solidify the Matrigel. The organoids were cultured in growth medium consisting of Ad-Df^+++^, B27 supplement (Invitrogen, 50×), N_2_ supplement (Invitrogen, 100×), *N*-acetylcysteine (Sigma-Aldrich, St. Louis, MO, USA,1 mM), A-83-01 (Tocris, Abingdon, United Kingdom, 0.5 μM), SB202190 (Selleck Chemicals, Houston, TX, USA, 10 μM), nicotinamide (Sigma-Aldrich, 10 mM), recombinant epidermal growth factor (EGF; Thermo Fisher, Waltham, MA, USA, 50 ng/ml), Noggin (Peprotech, Rocky Hill, NJ, USA, 100 ng/ml), and R-spondin 1–conditioned medium (10×) and Wnt3a-conditioned medium (2×), both produced in-house. Culture medium (IOM, 250 μl) supplemented with Y-27632 (Sigma-Aldrich, 10 μM) was added to each well, and plates were incubated at 37 °C. The culture medium was changed every second day and organoids passaged every 7 days. To attain cell differentiation, organoids were cultured in differentiation medium from which R-spondin 1, Wnt3a, SB201290, and nicotinamide were omitted. For single-cell re-plating experiments, intestinal organoids were cultured for 4 days and treated with TNF (10 ng/ml), with or without supplementation of PGE_2_ (1 μM), or treated with PGE_2_ alone for 48 h. Organoids cultured under these conditions were digested into single cells on day 7 in accordance with a well-established procedure [27]. Single cells (1500 cells) were cultured in IOM for 7 days, and the number of organoids was counted under a microscope on day 7. PGE_2_ was purchased from Cayman Chemical (Ann Arbor, MI, USA). Celecoxib (PZ0008) was bought from Sigma-Aldrich, and the organoids were treated with celecoxib (3 μM) for 48 h.

### 2.4 Mass Spectrometry

Primary monocytes from patients were incubated overnight, and the culture medium was subsequently replaced with serum-free RPMI 1640 basal medium. Cells were then treated with 10 ng/ml of TNF for 8 h. The medium was subsequently harvested and centrifuged at 13,000 ×*g* for 5 min at 4 °C. To quantify eicosanoids in the medium, 100 μl of medium was analyzed by gas chromatography–mass spectrometry (GC–MS analysis).

### 2.5 RNA Extraction, cDNA Synthesis, and qPCR

RNA was purified from intestinal organoids and monocytes using NucleoSpin columns (Macherey-Nagel, Düren, Germany). RNA (200 ng) was reverse transcribed into cDNA (SuperScript II reverse transcriptase, Invitrogen) followed by SYBR green (Roche, Basel, Switzerland) qPCR with β-actin as the housekeeping gene. All primer sequences are listed in the Supplemental Table S2.

## 3 RNA-seq Analysis

Intestinal organoids were cultured for 4 days and treated with TNF (10 ng/ml) with or without PGE_2_ (1 μM), or treated with PGE_2_ alone for 48 h. RNA was isolated (*n* = 3/group), and quality was assessed by Nanodrop (OD 260/280 ≥ 2.0 and OD 260/230 ≥ 2.0) and Agilent Bioanalyzer (RIN value ≥ 6.8). RNA-seq was done using 150-bp end reads at Novogene, Ltd. Data were deposited at GEO data set GSE 116936. Data normalization, statistical analysis, and hierarchical clustering were performed using Qlucore software (Qlucore, Lund, Sweden). Pathway and GO term analyses were performed using the Gene Ontology website (www.geneontology.org/).

## 4 Expression analyses in Independent Patient Cohorts

We analyzed *ANXA1* expression using transcriptomic data from an independent patient cohort. Intestinal biopsies were obtained form an independent patient cohort at Herlev Hospital consisting of healthy control subjects and UC patients with varying disease activity based on the Mayo score [23]. Two neighboring biopsies from inflamed sigmoid colon were obtained from each patient within a 5 cm radius, from which RNA was obtained and analyzed by microarray as previously described [28]. In addition, we analyzed *ANXA1* expression from Affymetrix microarray data downloaded from the Gene Expression Omnibus website (www.ncbi.nlm.nih.gov/geo/) and extracted data from the GSE14580 study [29]. The study consists of microarray expression data from intestinal biopsies from healthy control subjects, from Rs and PNRs before and after TNF inhibitor treatment in patients with UC. Data were generated using the Human Genome U133 Plus 2.0 array (Affymetrix) and normalized using robust multichip average (RMA), as described in the above-mentioned study entry.

### 4.1 Immunohistochemistry and Immunofluorescence

Colonic biopsies from the sigmoid part were obtained endoscopically from the patients before and after TNF inhibitor treatment. IHC for detection of COX-2 and MUC2 was performed on paraformaldehyde-fixed tissue and intestinal organoids. Sections were mounted on adhesive slides (Superfrost Plus, Menzel-Glaser, Braunschweig, Germany) and kept at 4 °C until staining on a DAKO autostainer (DAKO, Glostrup, Denmark). COX-2 (SP21) monoclonal antibody was purchased from Cell Marque (Rocklin, CA, USA). MUC2 (CCP58) antibody was purchased from DAKO. For immunofluorescent detection of MUC2 in intestinal organoids, deparaffinized slides were pretreated with citrate buffer (pH 6) in a DAKO antigen retrieval machine and then blocked with blocking buffer (3% BSA + 0.2% Triton-100) for 1 h at ambient temperature. Slides were incubated overnight at 4 °C with primary anti-MUC2 antibody diluted 1:200 in blocking buffer. Stained cells were washed with PBS (3 times, 10 min apart), followed by incubation with a secondary antibody (Goat anti-mouse-Alexa 488, Thermo Fisher) diluted 1:200 in blocking buffer for 1 h. DAPI (Thermo Fisher) was incubated for 5 min (0.1 mg/ml, diluted in PBS). After incubation, slides were washed with PBS (3 times, 10 min apart) and mounted using ProLong Gold Antifade Mountant (P10144, Thermo Fisher). All images were obtained with a Leica fluorescence microscope (Manalapan, NJ, USA). For periodic acid–Schiff (PAS) staining, paraformaldehyde-fixed intestinal organoids were mounted on adhesive slides, and PAS staining was carried out as described previously [30]. Mature goblet cells were quantified by counting the number of PAS^+^ goblet cells. Twenty-one microscopic images from PAS staining were counted for each condition. The percentage of goblet cells was defined as the number of PAS^+^ goblet cells compared with the total number of epithelial cells in each image.

## 5 Evaluation of COX-2 IHC Staining

COX-2 IHC scoring was conducted by the staff pathologist, who was blinded to all sample IDs. For each slide, the staining in lamina propria and epithelium was scored separately. The percentage of positive cells was scored as 0 (negative, no positive cells), 1 (<25%), 2 (25–50%), 3 (51–75%), and 4 (>75%). Staining intensity was graded as 0 (negative), 1 (weak), 2 (moderate), and 3 (strong). The degree of immunoreactivity was calculated as the sum of the intensity score and the fraction of positive cells.

### 5.1 Statistical Analysis

Data are presented using nonparametric statistics (i.e., medians with interquartile ranges). Comparisons between groups were completed using the Mann-Whitney *U* test. Multiple comparisons were performed using the Kruskal-Walls test: *P < 0.05; **P < 0.01; ***P < 0.001; ****P < 0.0001; ns = no statistical significant difference.

## 6 Results

### 6.1 Comparison of *COX-2* Expression and PGE_2_ Secretion between Responders and Primary Non-Responders

**Fig. 1:**
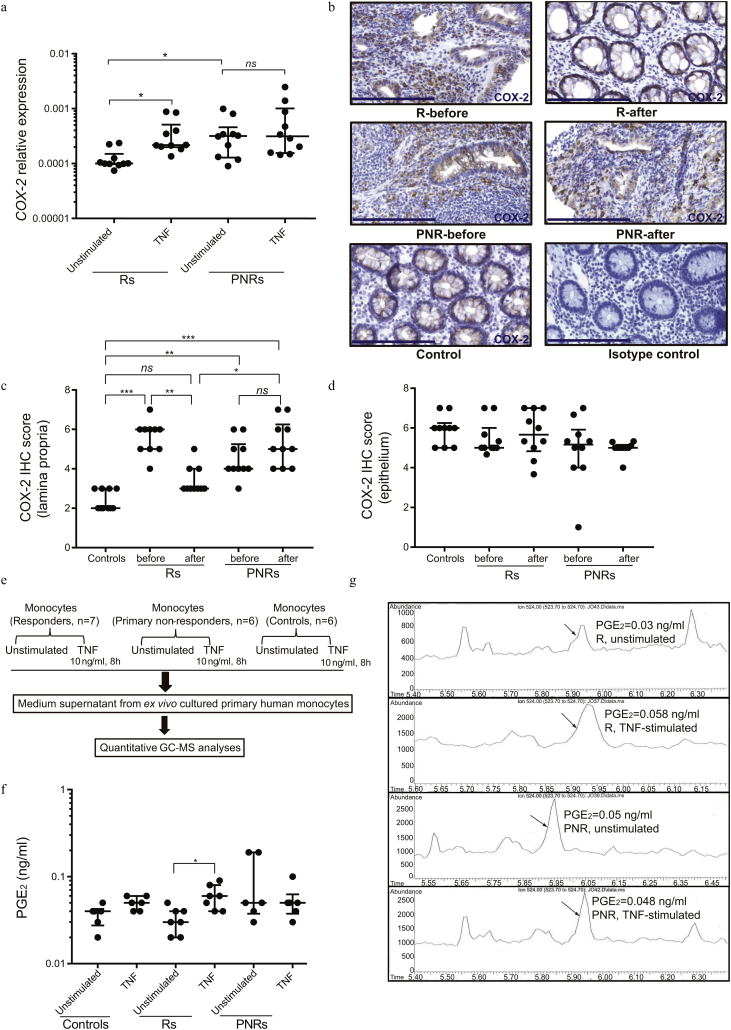
The COX-2–PGE_2_ pathway correlates with response to TNF inhibitor in primary monocytes. (a) Primary human monocytes were treated with TNF (10 ng/ml) for 8 h, followed by gene expression analysis of *COX-2* in responders (Rs) (n = 10) and primary non-responders (PNRs) (n = 10). (b) Representative images of COX-2 immunohistochemistry (IHC) stains of intestinal biopsies from Rs, PNRs (before and after TNF inhibitor treatment), healthy control subjects, and isotype control, counterstained by hematoxylin. Nuclei (blue), COX-2 (brown). Scale bar, 200 μm. Magnification, 40×. (c, d) Scoring of COX-2 abundance based on the IHC stains performed in lamina propria cells and epithelium of intestinal biopsies from Rs, PNRs (before and after TNF inhibitors treatment), and healthy control subjects. Rs, n = 10; PNRs, n = 10, and control subjects, n = 10. (e) The procedure applied for gas chromatography–mass spectrometry (GC–MS) analysis. (f) Protein levels of PGE_2_ were quantified by GC–MS analysis in the culture medium of primary human monocytes. Rs, n = 7; PNRs, n = 6; and control subjects, n = 6. (g) Chromatograms of PGE_2_ quantifications in the culture medium of primary monocytes. Representative images of one responder and one primary non-responder, with or without TNF stimulation, are depicted. (a, e, f, g) Primary monocytes were isolated from patients during maintenance therapy with a TNF inhibitor (Rs) or in case of PNRs following shift to a second-line therapy, e.g., vedolizumab. (b, c, d) Colonic biopsies were obtained from the patients before and after 14 weeks of TNF inhibitor induction therapy. (a, c, d, f) Data are shown as medians with interquartile ranges. The Kruskal-Walls test were used to compare the data. *P < 0.05; **P < 0.01; ***P < 0.001; ns = no statistically significant difference.

**Fig. S1:**
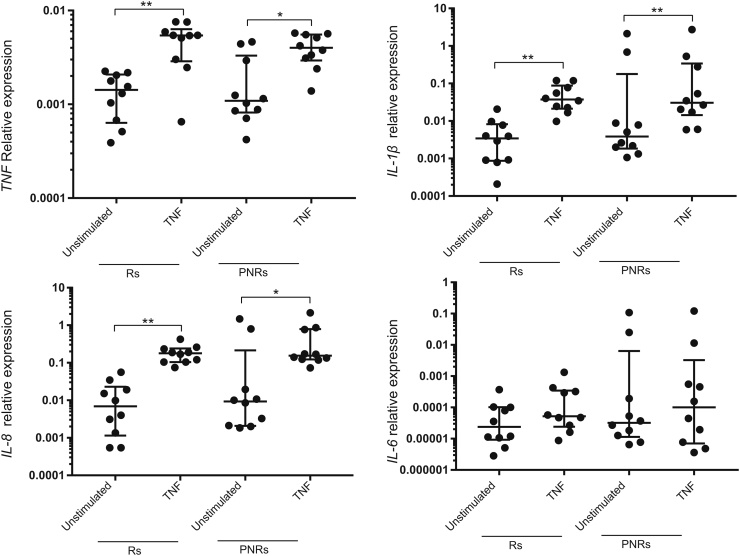
(correlated with Fig. 1). TNF caused a transcriptional induction of typical UC-associated cytokines in primary monocytes derived from responders (Rs) and primary non-responders (PNRs). Primary human monocytes were treated with TNF for 8 h, followed by gene expression analysis of *TNF*, *IL-1β*, *IL-8*, and *IL-6* in Rs (n = 10) and PNRs (n = 10). TNF treatment 10 ng/ml for 8 h. Data are shown as the median with interquartile ranges. The Kruskal-Walls test was used to compare the data. *P < 0.05; **P < 0.01; ***P < 0.001; ****P < 0.0001.

**Fig. S2:**
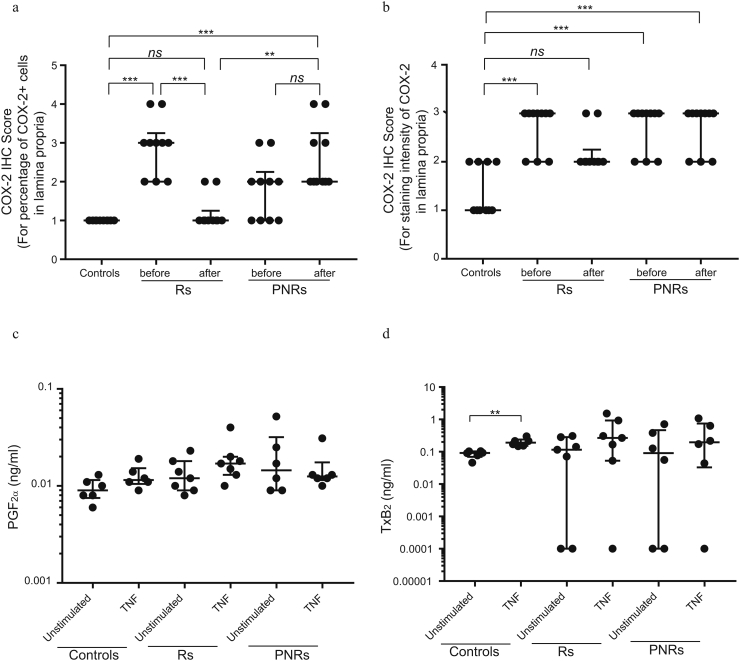
(correlated with Fig. 1). COX-2 IHC scoring in intestinal lamina propria and the quantified levels of PGF_2α_ and TxB_2_ quantified by GC–MS analysis. (a, b) Scoring of the percentage of COX-2^+^ cells (a) and scoring of the staining intensity of COX-2^+^ cells (b) based on the IHC stains performed in lamina propria cells from Rs, PNRs (before and after TNF inhibitor treatment), and control subjects Rs, n = 10; PNRs, n = 10, and control subjects, n = 10. (c) Protein levels of PGF_2α_ were quantified by GC–MS analysis. Rs (n = 7), PNRs (n = 6), and control subjects (n = 6). (d) Protein levels of TxB_2_ were quantified by GC–MS analysis. TxB_2_ can be detected in five Rs (n = 7), four PNRs (n = 6), and control subjects (n = 6). (a–d) Data are shown as the median with interquartile ranges. The Kruskal-Walls test was used to compare the data. *P < 0.05; **P < 0.01; ***P < 0.001; ****P < 0.0001; ns = no statistically significant difference.

Given that monocytes are important drivers of intestinal inflammation [9,31], we assessed the response of primary monocytes isolated from Rs and PNRs to TNF stimulation. TNF caused a prominent transcriptional induction of typical UC-associated cytokines (*TNF*, *IL-1β*, *IL-8*, and *IL-6*), which was comparable between Rs and PNRs (Fig. S1). TNF stimulation of primary monocytes from Rs was, however, associated with a twofold induction of *COX-2* (*PTGS2*) expression (*P* < 0.01), while no such induction was observed in monocytes from PNRs (Fig. 1a). Importantly, the basal expression level of *COX-2* in unstimulated monocytes from PNRs was similar to the level observed in TNF-stimulated monocytes from Rs (Fig. 1a). Monocytes from PNRs therefore appeared to be maintained in an “inflammatory” state with respect to *COX-2* expression. In intestinal colonic biopsies from Rs and PNRs (both before and after TNF inhibitor treatment) and from healthy control subjects, COX-2 was detected in both epithelial cells and cells of the lamina propria. In the epithelial cells, the expression of COX-2 showed no significant changes under different conditions (Fig. 1d). Interestingly, COX-2 expression in the lamina propria correlated with ongoing and unresolved inflammation in PNRs following TNF inhibitor treatment (Fig. 1b, c, Fig. S2a, b). Thus, in vitro TNF stimulation led to the induction of *COX-2* in Rs, and there was a higher expression level of *COX-2* in monocytes from PNRs in the absence of exogenous TNF. In vivo, anti-TNF treatment can resolve the inflammation and reduce COX-2’s expression in the lamina propria of Rs but not in PNRs.

To determine the functional consequences of enhanced basal *COX-2* expression in monocytes from PNRs, prostanoid levels were measured in culture medium by mass spectrometry (Fig. 1e). Levels of PGE_2_, PGF_2α_, and TxB_2_ (the stable metabolite of TxA_2_) were quantifiable, whereas PGI_2_ and PGD_2_ were undetectable (Fig. 1f, g and Fig. S2c, d). Consistent with the TNF-mediated induction of *COX-2* expression in monocytes derived from Rs (Fig. 1a), the level of PGE_2_ was elevated following TNF stimulation of monocytes from Rs but not in monocytes from PNRs (Fig. 1f, g). The basal PGE_2_ levels were again trending toward higher levels in PNRs compared with Rs (*P* = 0.06). No differences in levels of PGF_2α_ or TxB_2_ between Rs or PNRs before or after TNF stimulation was detected (Fig. S2c, d). Accordingly, PGE_2_ production was directly affected by the abnormal regulation of COX-2 in PNRs. Of notice, no correlation between patients' baseline Mayo score and the basal level of *COX2* expression/PGE_2_ production was detected (Supplemental Table S3 and Table S4).

### 6.2 Transcriptional Profiling of Intestinal Organoids Stimulated with TNF and PGE_2_

**Fig. 2:**
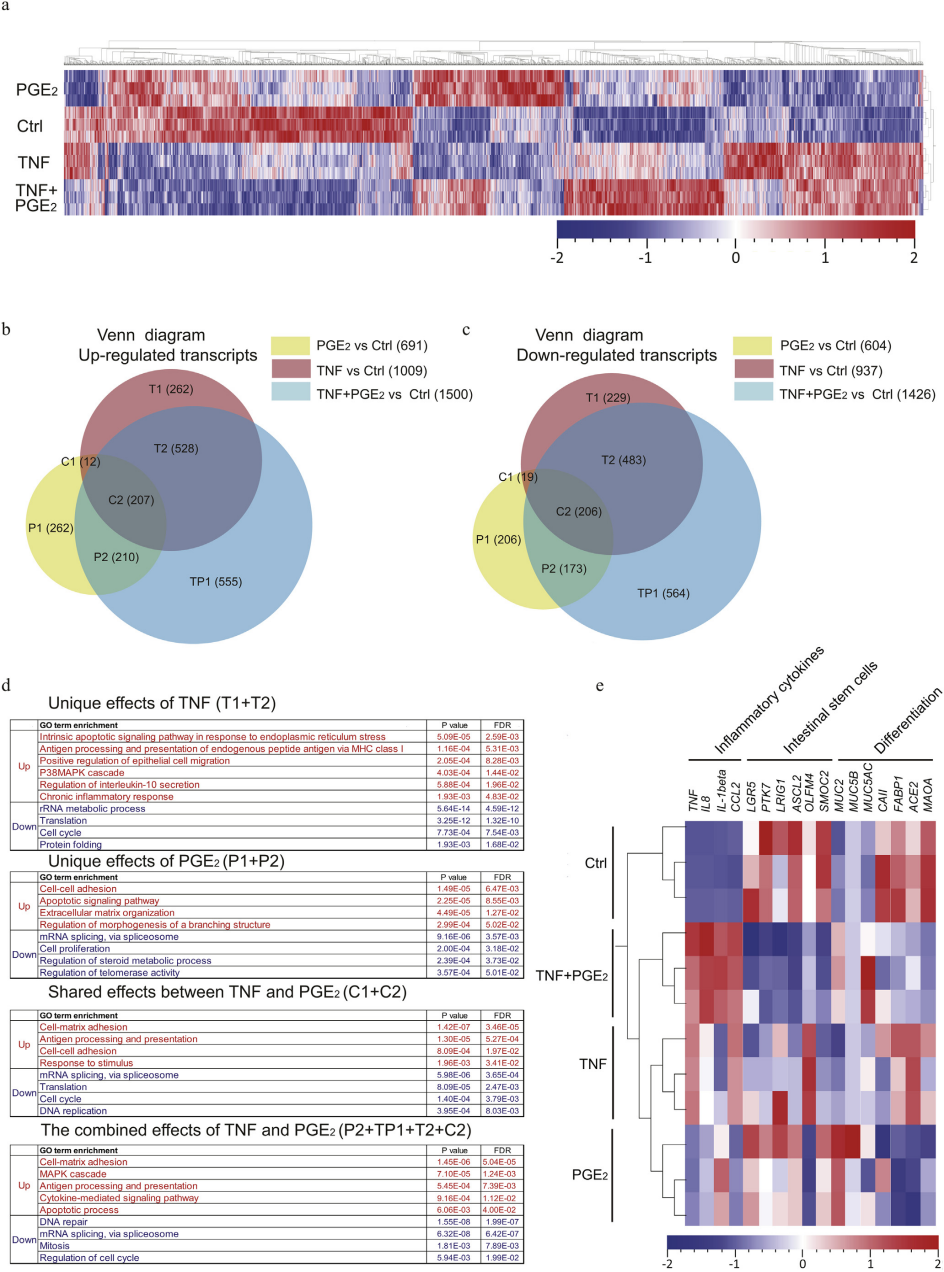
Transcriptomic profiling of organoids stimulated with TNF and PGE_2_. (a) Organoids cultured in IOM were treated for 48 h with TNF (10 ng/ml), PGE_2_ (1 μM) or their combination. Transcript levels from RNA sequencing were converted into z-scores and represented as a heatmap with hierarchical clustering of all significantly different expressed transcripts (4905; P < 0.01) between the 4 groups (n = 3 samples for each group). Rows represent samples whereas columns represent genes. Red and blue indicates transcripts with increased or decreased expression levels based on z-score transformation. (b-c) The stimulated conditions were compared to control (fold-change ≥2 and P < 0.01), and the deregulated transcripts following stimulation with TNF (1946 transcripts), PGE_2_ (1295 transcripts) and TNF + PGE_2_ (2926 transcripts) were identified. The deregulated genes of the three stimulated groups were divided into up- and down-regulated genes and presented as Venn diagrams. (d) The gene ontology (GO) biological process term analysis which show the pathways associated with up-regulated and down-regulated genes. The GO biological processes were organized by *P*-Value and False Discovery Rate (FDR) < 0.05. (e) Heatmap presenting the transcription profile of selected genes. These are markers for inflammatory cytokines (*TNF, IL8, IL-1beta, and CCL2*), intestinal stem cells (*LGR5, PTK7, LRIG1, ASCL2, OLFM4,* and *SMOC2*) and differentiation (*MUC2, MUC5B, MUC5AC, CAII, FABP1, ACE2, and MAOA*) in the 4 different conditions. Red and blue indicates transcripts with increased or decreased expression based on z-scores normalization.

To address the effect of inflammatory stimulation on intestinal epithelial cells, we performed expression analysis by RNA-seq on human colonic epithelial organoids treated with TNF and/or PGE_2_. The analysis revealed 4905 differentially expressed transcripts upon stimulation with TNF, PGE_2_ or both with each culture condition exhibiting unique sets of up and down regulated transcripts (*P* < 0.01; Fig. 2a). The 1946, 1295 and 2926 transcripts specifically deregulated by TNF, PGE_2_ and the combination when compared to controls (fold change ≥2, P < 0.01), were divided into up- and down-regulated genes exhibiting both unique and overlapping transcriptional responses to the treatments (Fig. 2b, c).

To elucidate the functional consequences of the stimulation (TNF, PGE_2_, or the combination), the biological processes associated with the differentially expressed transcripts were assessed by gene ontology (GO) enrichment analysis (Fig. 2d) (FDR < 0.05). The analyses revealed that uniquely up-regulated transcripts following TNF stimulation (T1 + T2 in Fig. 2b) were enriched for biological processes related to general inflammation, including apoptosis, antigen presentation, IL-10 secretion, chronic inflammatory response as well as epithelial cell migration (Fig. 2d). The transcripts uniquely up-regulated by PGE_2_ (P1 + P2 in Fig. 2b) were similarly associated with cellular apoptosis, and additionally enriched in pathways associated with extracellular matrix organization, cell-cell adhesion and regulation of a branching structure (Fig. 2d). The shared up-regulated effects between TNF and PGE_2_ (C1 + C2 in Fig. 2b) were enriched for cell adhesion and inflammation related antigen processing and presentation (Fig. 2d). The up-regulated transcripts when combining TNF and PGE_2_ (P2 + TP1 + T2 + C2 in Fig. 2b) were associated with inflammation resembling the biological functions of TNF stimulation with an additional impact on processes related to cell-matrix adhesion (Fig. 2d). All the down-regulated transcripts (Fig. 2c) were mainly correlated to basic cellular processes, including DNA replication, RNA processing, translation and cell proliferation, with no major differences in the associated GO-term between the treatment conditions (Fig. 2d).

The expression of inflammatory cytokines and marker genes for specific cellular subsets were additionally evaluated (Fig. 2e). As expected, TNF upregulated the expression of inflammatory cytokines, which could be further enhanced by the addition of PGE_2_. Moreover, repression of intestinal stem cell markers by PGE_2_ following TNF stimulation was observed, implying a synergistic effect of TNF and PGE_2_ stimulation on both inflammation and stem cell maintenance. In addition, we found that the expression of goblet cell markers (*MUC2, MUC5B,* and *MUC5AC*) was induced by PGE_2_, and that PGE_2_ downregulated the expression of enterocytes markers (*CAII, FABP1, ACE2, and MAOA*). These data combined indicate that continuous activation of the COX-2-PGE_2_ pathway in PNRs exacerbates the proinflammatory state of intestinal epithelium induced by TNF, thus potentially reducing the stem cell population and impairing epithelial regeneration.

### 6.3 Effect of PGE_2_ on the Self-Renewal of Intestinal Epithelial Stem Cells

**Fig. 3:**
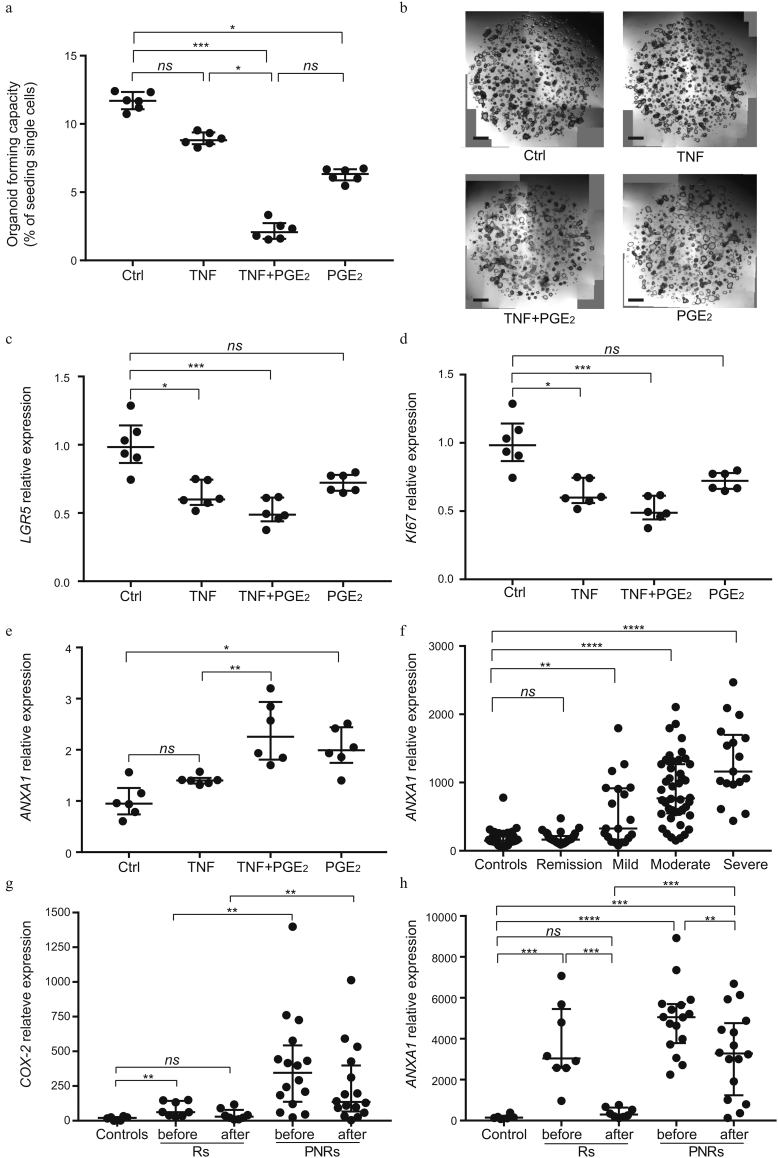
PGE_2_ and TNF affect the maintenance of intestinal stem cells. (a) Organoids cultured in IOM were treated for 48 h with a combination of TNF (10 ng/ml) and PGE_2_ (1 μM). Single-cell re-plating was performed on day 7, and cells were cultured in IOM medium for an additional 10 days. The organoid-forming capacity was assessed on day 7. (b) Microscopic images of intestinal organoids on day 7 before reseeding of single cells, described in (a). Scale bar, 100 μm. (c–e) Organoids cultured in IOM were treated with combinations of TNF (10 ng/ml) and PGE_2_ (1 μM) for 48 h, followed by gene expression analysis of *LGR5*, *KI67*, and *ANXA1*. (f) Gene expression of *ANXA1* from microarray data on colonic tissues of patients with active UC (mild: n = 11; moderate: n = 24; and severe: n = 10), UC in remission (n = 21), and healthy control subjects (n = 20). Data are shown as medians with interquartile ranges. The Kruskal-Walls test was used to compare the data. (g, h) Gene expression of *COX-2*/*ANXA1* from previous microarray data on colonic tissues from healthy control subjects (n = 6), Rs (n = 8), and PNRs (n = 16) including values before and after treatment with infliximab. Data were derived from GEO data set GSE14580. (a, c-e) Data are shown as the median of six biological replicates with interquartile ranges and are from one of three independent experiments in which the organoids were from three different patients. The Kruskal-Walls test was used to compare the data. *P < 0.05; **P < 0.01; ***P < 0.001; ****P < 0.0001; ns = no statistically significant difference.

**Fig. S3:**
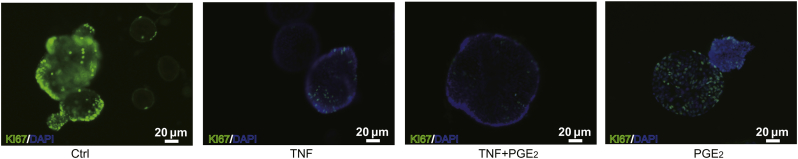
(Correlated with Fig. 3). KI67 staining of intestine organoids under different culture conditions. Organoids cultured in IOM were treated with combinations of TNF (10 ng/ml) and PGE_2_ (1 μM) for 48 h, and subsequence immunofluorescence was performed for the detection of KI67. DAPI (blue), KI67 (green). Scale bar, 20 μm.

With the identified impact of PGE_2_ on the expression of markers of stem cells and the reported beneficial effect on in vitro expansion of intestinal epithelial cells [32], we performed single-cell reseeding experiments in order to measure the direct effect of PGE_2_ on the fraction of cells with self-renewal properties. Whereas pretreatment with TNF alone did not significantly alter organoid-forming capacity, the combination with PGE_2_ led to a significant reduction in organoid-forming efficiency when compared with control samples (P < 0.001; Fig. 3a, b). Despite the previously reported role of PGE_2_ as supporting long-term maintenance of intestinal epithelial cells [32], treatment with PGE_2_ alone reduced the organoid-forming efficiency when compared with unstimulated cells (Fig. 3a, b). In line with the reduced self-renewal capacity and the observed reduction in the expression of stem cell marker genes in the RNAseq experiments, organoids treated with TNF and PGE_2_ also expressed lower levels of the adult stem cell marker *LGR5* and the proliferation marker *KI67* when compared with unstimulated control samples (Fig. 3c, d, Fig. S3). These findings show that cells in a TNF and PGE_2_ driven proinflammatory state have a compromised stem cell function. Of note, Annexin A1 (*ANXA1*), which is expressed specifically by the repairing epithelium following tissue damage [33], was upregulated on PGE_2_ stimulation suggesting that this is associated with a change in cellular identity (Fig. 3e). Analysis of microarray expression profiles obtained from intestinal biopsies of patients with active UC and healthy control subjects demonstrated a significant correlation between *ANXA1* expression and disease severity (Fig. 3f). Additionally, previously published expression data (GSE14580) of intestinal biopsies from patients with UC [29], including Rs and PNRs to TNF inhibitors, showed that the expression of both *COX-2* and *ANXA1* indeed are elevated in PNRs when compared with Rs and healthy control subjects (Fig. 3g, h). These observations indicate that a higher expression of *COX-2* and *ANXA1* accompanies a continued inflammatory state in PNRs following therapy with TNF inhibitors.

### 6.4 Effect of PGE_2_ on the Differentiation of Intestinal Epithelial Stem Cells

**Fig. 4:**
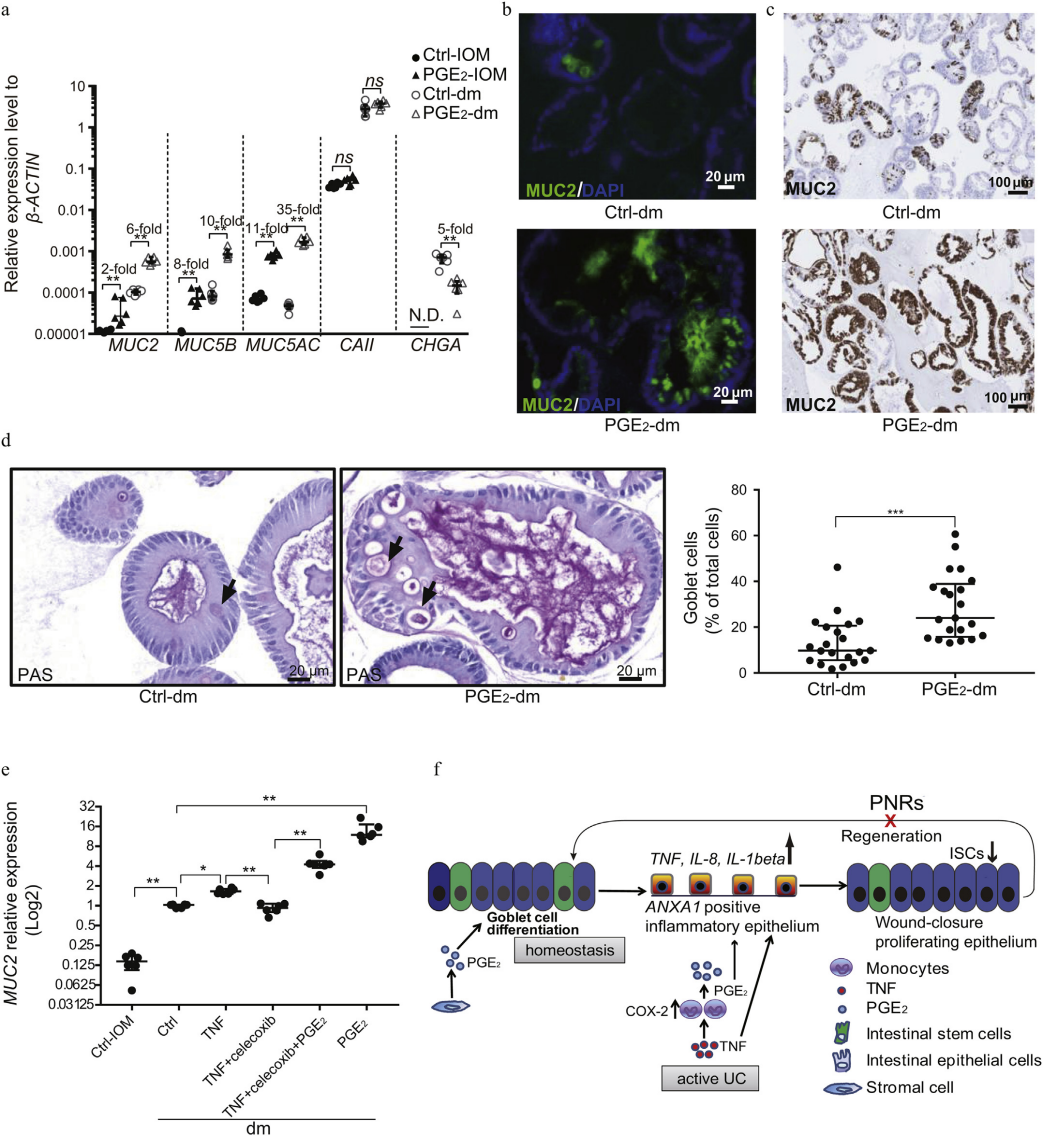
PGE_2_ promotes the expression of mucins. (a) Organoids were cultured in IOM or dm, with or without the addition of PGE_2_ for 48 h. PGE_2_ treatment (1 μM). Gene expression of mucins (*MUC2*, *MUC5B*, and *MUC5AC,* which are markers for goblet cells), *CAII* (enterocyte marker), and *CHGA* (enteroendocrine marker) were measured by qPCR. The expression of *CHGA* could not be detected in IOM culture conditions. N.D. = not detected. (b–d) Organoids cultured in dm were treated with or without PGE_2_ for 48 h, and then immunofluorescence (b) and IHC (c) were performed for detection of MUC2. DAPI (blue), MUC2 (green). Periodic acid–Schiff (PAS) staining (d) was conducted, and mature goblet cells were quantified by counting of PAS^+^ goblet cells. (e) Organoids cultured in dm were treated with combinations of TNF (10 ng/ml), the COX-2 inhibitor celecoxib (3 μM), and PGE_2_ (1 μM) for 48 h. Gene expression of *MUC2* was assessed by qPCR. (f) Schematic depiction of the correlation between COX-2–PGE_2_ pathway and the responsiveness to treatment with TNF inhibitors. (a, e) Data are shown as the median of six biological replicates with interquartile ranges and are from one of three independent experiments. The Mann-Whitney *U* test was used to compare the data. (d) Data are shown as the median with interquartile ranges and are from one of two independent experiments. Mann-Whitney *U* tests were used to compare the data. *P < 0.05; **P < 0.01; ***P < 0.001; ns = no statistically significant difference.

**Fig. S4:**
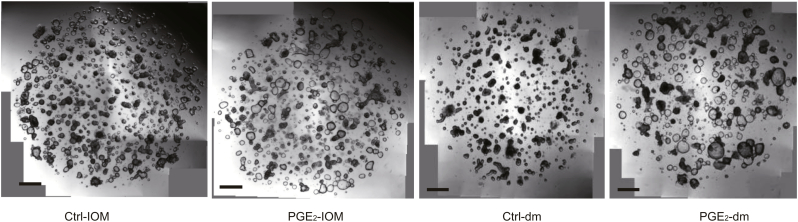
(Correlated with Fig. 4a). Microscopic images of intestinal organoids under different culture conditions. Organoids were cultured in IOM or d_m_, with or without the addition of PGE_2_ for 48 h. All images were obtained with a Leica microscope. Scale bar, 100 μm.

Appropriate differentiation of the intestinal epithelium is crucial following inflammation and wound formation for reestablishing a functional epithelial barrier [34]. We therefore explored the effects of PGE_2_ on intestinal epithelial differentiation by altering medium composition from conditions supporting stem cell self-renewal and expansion (intestinal organoid medium [IOM]) to differentiation (differentiation medium [dm]) (Fig. S4). Under IOM conditions, stimulation with PGE_2_ induced a slight increase in the expression of several mucin-associated genes normally expressed by goblet cells (MUCIN2 [*MUC2*] [twofold, P < 0.01], *MUC5B* [eightfold, P < 0.01], *MUC5AC* [11-fold, P < 0.01]) (Fig. 4a), confirming the effects on goblet cell differentiation previously observed (Fig. 2e). PGE_2_ stimulation did not affect the expression of carbonic anhydrase II (*CAII*) or chromogranin A (*CHGA*), which are markers for enterocytes and enteroendocrine cells, respectively (Fig. 4a). Following culture of the cells under differentiation conditions, which mimic the egress of stem cells from the stem cell compartment, we found that PGE_2_ dramatically induced the expression of markers for goblet cells (*P* < 0.01; Fig. 4a), where expression of *CHGA* was suppressed (Fig. 4a, Supplemental Table S5). At the protein level, MUC2 (the dominant gel-forming mucin produced by goblet cells) was markedly induced by PGE_2_, as determined by both fluorescent and chromogenic IHC staining of organoid cultures (Fig. 4b, c). Moreover, this was associated with a concordant increase in the number of PAS^+^ cells, which represents a hallmark of goblet cell differentiation (Fig. 4d). Using the specific COX-2 inhibitor celecoxib, the induction of *MUC2* expression by TNF was inhibited, and the inhibitory effect of celecoxib could be bypassed by addition of exogenous PGE_2_ (Fig. 4e).

In this way, our data suggest that the effect of PGE_2_ on intestinal epithelium depends on the presence of concomitant inflammation. During intestinal homeostasis, PGE_2_ promotes goblet cell differentiation, whereas it drives the epithelial cells into a state with impaired stem cell population during inflammation (Fig. 4f).

## 7 Discussion

Identification of mechanisms that influence the responsiveness to TNF inhibitors might enable clinicians to predict the response and to potentially devise new and more rational and tailored treatment strategies. This is consequently of major clinical importance for the management of patients with IBD. In this study, we for the first time show that monocytes from Rs display increased expression of *COX-2* following TNF stimulation, whereas this is not the case for PNRs due to a higher basal expression level of *COX-2*. The continuously augmented inflammation-independent expression of *COX-2* and secretion of PGE_2_ by monocytes from PNRs, indicate that monocytes from PNRs fail to regulate PGE_2_ synthesis, which potentially leads to exacerbation of the inflammatory state in the intestinal mucosa. This notion is supported by the observation that the dominant metabolite induced by COX-2, PGE_2_, in this experimental setup enhances the proinflammatory effect of TNF in intestinal epithelial cells.

The PNRs in this study were initially treated with a TNF inhibitor, infliximab, but were after the induction regimen switched out of class to different therapeutic options due to the lack of response. These therapeutic options include anti-integrins such as vedolizumab, along with e.g., thiopurines like azathioprine, glucocorticoids or 5-ASA. COX-2 expression has been proposed to be targeted by glucocorticoids, which was administered to one out of ten PNRs in this study [35]. Furthermore, seven out of ten PNRs received vedolizumab therapy at blood sampling. However, it remains unknown whether vedolizumab affects the level of COX-2 in monocytes. Moreover, the use of azathioprine and 5-ASA in the study cohort was comparable between Rs and PNRs (Supplemental Table S1). These two therapies might also influence the expression of COX-2 as previously reported in DSS-induced murine colitis and in cancer cell lines [36,37]. Although the regulation of COX-2 could be affected by the therapies, the patient cohorts analyzed in this study were largely comparable except for the biologics with scarcely reported impact on COX-2 activity and levels. Thus, our results therefore reveal that it might be of importance to further validate the expression of COX-2 prior to treatment with TNF inhibitors in the future.

Induction of COX-2 has been shown to correlate with initiation of the mucosal healing process following intestinal wound formation [16,17,38], and the concentration of PGE_2_ is elevated in colonic tissue of patients with flaring UC [39]. In the initial phases of wound healing, murine studies have revealed that PGE_2_ induces a specific cellular state characterized by the expression of genes associated with WAE cells [19]. It is evident that induction of PGE_2_ and the existence of WAE cells is transient, and once wound closure has been achieved, PGE_2_ levels return to normal [40]. Epithelial re-differentiation will subsequently direct completion of tissue remodeling and restore of epithelial integrity. Taken together, these results imply that PGE_2_ plays a key role in the initial phases of wound re-epithelialization. We demonstrate that ongoing COX-2 activity precludes later phases of regeneration and ultimately mucosal healing. The expression analysis supports this notion, as a GO analysis reveals that biological processes regulated by PGE_2_ are functionally associated with cell-cell adhesion and cellular apoptosis, and able to exacerbate the proinflammatory state of intestinal epithelial cells following stimulation with TNF.

In terms of cellular differentiation, PGE_2_ induced the expression of markers for goblet cells both during homeostasis, but also during TNF induced inflammation. The importance of PGE_2_ for goblet cell differentiation during steady-state homeostasis is reinforced by the clinical observations indicating a central role of COX-2 for the maintenance of remission in UC [16,17]. Further, we observed that PGE_2_ inhibited the self-renewal of intestinal stem cells during TNF treatment. In fact, the combination of PGE_2_ and TNF represses normal stem cell functions and drives cells into a different inflammatory state that is characterized by expression of *TNF*, *IL-8*, *IL-1β*, and *AXNA1*. Hence our findings indicate that in PNRs, the sustained induction of PGE_2_ continuously promotes cell states associated with the inflammatory epithelium, thereby compromising tissue remodeling and regeneration. Epithelial reconstitution is a pivotal treatment goal in UC, a process in which both epithelial cells and monocytes/macrophages are involved [9,31]. Unlike Rs, where monocytes upon treatment with TNF inhibitors decrease *COX-2* expression and thus reduce PGE_2_ secretion, the epithelial cells of PNRs are kept in an inflammatory state, which in turn, is incompatible with reestablishment of tissue homeostasis. Thus, we propose a novel pathophysiologic mechanism based on PGE_2_ and TNF in the epithelial injury response associated with flaring UC, and we provide a plausible explanation as to why PNRs do not benefit from treatment with TNF inhibitors. We also describe for the first time an important and previously overlooked function of PGE_2_ in regulating the differentiation of human intestinal epithelial cells during homeostatic conditions by promoting barrier reinforcement via goblet cell differentiation and mucin production.

Although this study was not prospective in its design, it is tempting to speculate that the basal expression of *COX-2* in monocytes may be used to identify Rs and PNRs before initiation of TNF inhibitors in future clinical settings. A better understanding of PGE_2_ signaling and its impact on the different phases of tissue homeostasis and regeneration will help to shed light on the underlying mechanisms of the COX-2–PGE_2_ axis, and whether this might be a druggable pathway to modulate TNF responsiveness in the future.

The following are the supplementary data related to this article.

Supplemental Table S1 Clinical characteristics of patients with ulcerative colitis. Supplemental Table S1

Supplemental Table S2 List  of PCR primers applied in this study. Supplemental Table S2

Supplemental Table S3 The correlations between the patients' clinical Mayo Score and the expression of *COX-2* in primary monocytes at unstimulated conditions of Figure 1a. Supplemental Table S3

Supplemental Table S4 The correlations between the patients' clinical Mayo Score and the protein level of PGE2 in the cultured medium of primary monocytes at unstimulated conditions of Figure 1f. Supplemental Table S4

Supplemental Table S5 Gene expression values relative to *β-ACTIN* in Figure 4a. Supplemental Table S5

## Funding sources

K.B.J. was supported by grants from the Novo Nordisk Foundation (NNF17CC0027852) and the Lundbeck Foundation (R105-A9755). D.M.A. was supported by a Pilot and Feasibility grant from the Vanderbilt Digestive Disease Research Center and the National Institutes of Health (NIH) Grants DK058404 and AI121796. Y.L. was supported by Aase and Ejnar Danielsen's Foundation, Denmark (F-19215-00-27) and the A.P. Møller Foundation.
