# Supplementary material for: COX-2–PGE2 Signaling Impairs Intestinal Epithelial Regeneration and Associates with TNF Inhibitor Responsiveness in Ulcerative Colitis
Source: eBioMedicine. 2018 Sep 3;36:497–507. doi: 10.1016/j.ebiom.2018.08.040 (PMC6197735; doi:10.1016/j.ebiom.2018.08.040)
Supplement: Supplemental Table S1 — Clinical characteristics of patients with ulcerative colitis. [file mmc1.docx]

**Supplemental Table S1 Clinical characteristics of patients with ulcerative colitis**

| **Clinical variables** | **Responders**  n=10 | **Primary non-responders**  n=10 |
| --- | --- | --- |
| Gender (male/female) | 8/2 | 6/4 |
| Age, years (median, IQR) | 41·5 (23·25-52·5) | 40·5 (32-47·75) |
| Clinical Mayo score (median, IQR) when blood samples were drawn for the project | 0 (0-2·75) | 0 (0-3·25) |
| Mayo score (median, IQR) week 0 (before biologics) | 9 (7·75-10) | 11(9·5-11) |
| Mayo score (median, IQR)  at week 14 | 1 (0-3·25) | 9 (7·75-10) |
| Endoscopic subscore (median, IQR) week 0 (before biologics) | 2·5 (2-3) | 3 (2-3) |
| Endoscopic subscore (median, IQR) at week 14 | 0 (0-1) | 2 (0·75-3)* |
| Past anti-TNF exposure (yes/no) | 10/0 | 10/0 |
| **Daily medication** | | |
| -Azathioprine | 4 | 1 |
| -Steroids | 0 | 1 |
| -5-ASA | 9 | 8 |
| -Anti-TNF exposure | 7 | 0 |
| -Vedolizumab | 0 | 7 |

For the responders and primary non-responders, the clinical Mayo score was recorded when blood samples were drawn. 5-ASA, 5-aminosalicylic acid. IQR, interquartile range. * Two patients (PNRs) were colectomized.
