# Supplementary material for: COX-2–PGE2 Signaling Impairs Intestinal Epithelial Regeneration and Associates with TNF Inhibitor Responsiveness in Ulcerative Colitis
Source: eBioMedicine. 2018 Sep 3;36:497–507. doi: 10.1016/j.ebiom.2018.08.040 (PMC6197735; doi:10.1016/j.ebiom.2018.08.040)
Supplement: Supplemental Table S2 — List of PCR primers applied in this study. [file mmc2.docx]

**Supplemental Table S2**

| human *β-ACTIN* F1 | 5'CATGTACGTTGCTATCCAGGC3' |
| --- | --- |
| human *β-ACTIN* R1 | 5'CTCCTTAATGTCACGCACGAT3' |
| human *COX-2*-F1 | 5'CTGGCGCTCAGCCATACAG3' |
| human *COX-2*-R1 | 5'CGCACTTATACTGGTCAAATCCC3' |
| human *TNF*-F1 | 5'ATGAGCACTGAAAGCATGATC3' |
| human *TNF*-R1 | 5'GAGGGCTGATTAGAGAGAGGT3' |
| human *IL-8*-F1 | 5'TTTTGCCAAGGAGTGCTAAAGA3' |
| human *IL-8*-R1 | 5'AACCCTCTGCACCCAGTTTTC3' |
| human *IL-1β*-F1 | 5'CTCGCCAGTGAAATGATGGCT3' |
| human *IL-1β*-R1 | 5'GTCGGAGATTCGTAGCTGGAT3' |
| human *IL-6*-F1 | 5'ACTCACCTCTTCAGAACGAATTG3' |
| human *IL-6*-R1 | 5'CCATCTTTGGAAGGTTCAGGTTG3' |
| human *IL-18*-F1 | 5'TCTTCATTGACCAAGGAAATCGG3' |
| human *IL-18*-R1 | 5'TCCGGGGTGCATTATCTCTAC3' |
| human *MUC2*-F1 | 5'AGGATGACACCATCTACCTCAC3' |
| human *MUC2*-R1 | 5'CATCGCTCTTCTCAATGAGCA3' |
| human *MUC5B*-F1 | 5'GCCTACGAGGACTTCAACGTC3' |
| human *MUC5B*-R1 | 5'CCTTGATGACAACACGGGTGA3' |
| human *MUC5AC*-F1 | 5'TCTGCATCGATTGGAGAGGC3' |
| human *MUC5AC*-R1 | 5'TAGCAGTAGGAGGGGTTGCT3' |
| human *CAII*-F1 | 5'GGGTACGGCAAACACAACG3' |
| human *CAII*-R1 | 5'GGCTGTATGAGTGTCGATGTC3' |
| human *CHGA*-F1 | 5'TAAAGGGGATACCGAGGTGATG3' |
| human *CHGA*-R1 | 5'TCGGAGTGTCTCAAAACATTCC3' |
| human *ANXA1*-F1 | 5'GCGGTGAGCCCCTATCCTA3' |
| human *ANXA1-*R1 | 5'TGATGGTTGCTTCATCCACAC3' |

List of qPCR primers
