# Supplementary material for: COX-2–PGE2 Signaling Impairs Intestinal Epithelial Regeneration and Associates with TNF Inhibitor Responsiveness in Ulcerative Colitis
Source: eBioMedicine. 2018 Sep 3;36:497–507. doi: 10.1016/j.ebiom.2018.08.040 (PMC6197735; doi:10.1016/j.ebiom.2018.08.040)
Supplement: Supplemental Table S3 — The correlations between the patients' clinical Mayo Score and the expression of COX-2 in primary monocytes at unstimulated conditions of Figure 1a. [file mmc3.docx]

**Supplemental Table S3**

**The correlations between the patients’ clinical Mayo Score and the expression of *COX-2* in primary monocytes at unstimulated conditions of Figure 1a.**

| Rs | Clinical Mayo score | COX-2 basal mRNA level | PNRs | Clinical Mayo score | COX-2 basal mRNA level |
| --- | --- | --- | --- | --- | --- |
| pt. 45 | 1 | 0·000098 | pt. 46 | 0 | 0·000118322 |
| pt. 50 | 0 | 0·000225435 | pt. 47 | 2 | 0·000804288 |
| pt. 51 | 0 | 0·000074 | pt. 48 | 0 | 0·000089 |
| pt. 52 | 1 | 0·0000994 | pt. 56 | 4 | 0·000342882 |
| pt. 53 | 9 | 0·000093 | pt. 57 | 0 | 0·000323264 |
| pt. 54 | 0 | 0·000237465 | pt. 58 | 5 | 0·000132199 |
| pt. 55 | 0 | 0·000099 | pt. 59 | 0 | 0·000216252 |
| pt. 60 | 0 | 0·000105169 | pt. 63 | 0 | 0·000312252 |
| pt. 61 | 0 | 0·000125068 | pt. 64 | 0 | 0·00099 |
| pt. 62 | 8 | 0·000100885 | pt. 65 | 3 | 0·000341 |
| Correlation | P=0·4 R^2^=0·09 | | Correlation | P=0·8 R^2^=0·008 | |

For the responders and primary non-responders, the Mayo score was recorded and blood samples were obtained after week 14. Higher clinical Mayo score does not correlate with the elevated *COX-2* expression. This indicates that the mRNA level of *COX-2* differences is not affected by disease activity.
