# Supplementary material for: COX-2–PGE2 Signaling Impairs Intestinal Epithelial Regeneration and Associates with TNF Inhibitor Responsiveness in Ulcerative Colitis
Source: eBioMedicine. 2018 Sep 3;36:497–507. doi: 10.1016/j.ebiom.2018.08.040 (PMC6197735; doi:10.1016/j.ebiom.2018.08.040)
Supplement: Supplemental Table S4 — The correlations between the patients' clinical Mayo Score and the protein level of PGE2 in the cultured medium of primary monocytes at unstimulated conditions of Figure 1f. [file mmc4.docx]

**Supplemental Table S4**

**The correlations between the patients’ clinical Mayo Score and the protein level of PGE2 in the cultured medium of primary monocytes at unstimulated conditions of Figure 1f.**

| Rs | Clinical Mayo score | PGE2 basal level | PNRs | Clinical Mayo score | PGE2 basal level |
| --- | --- | --- | --- | --- | --- |
| pt. 50 | 0 | 0·03 | pt. 47 | 2 | 0·052 |
| pt. 51 | 0 | 0·02 | pt. 48 | 0 | 0·025 |
| pt. 52 | 1 | 0·04 | pt. 57 | 0 | 0·009 |
| pt. 53 | 9 | 0·04 | pt. 58 | 5 | 0·009 |
| pt. 60 | 0 | 0·05 | pt. 59 | 0 | 0·012 |
| pt. 61 | 0 | 0·03 | pt. 63 | 0 | 0·017 |
| pt. 62 | 8 | 0·02 |  |  |  |
| Correlation | P=0·54 R^2^=0·08 | | Correlation | P=0·96 R^2^=0·00066 | |

After week 14, the Mayo score was recorded and blood samples were drawn from responders and primary non-responders. Higher clinical Mayo score does not lead to a higher PGE_2_ production. This indicates that the differences of PGE_2_ production is not caused by differences of disease severity among patients.
