# Supplementary material for: COX-2–PGE2 Signaling Impairs Intestinal Epithelial Regeneration and Associates with TNF Inhibitor Responsiveness in Ulcerative Colitis
Source: eBioMedicine. 2018 Sep 3;36:497–507. doi: 10.1016/j.ebiom.2018.08.040 (PMC6197735; doi:10.1016/j.ebiom.2018.08.040)
Supplement: Supplemental Table S5 — Gene expression values relative to β-ACTIN in Figure 4a. [file mmc5.docx]

**Supplemental Table S5 Gene expression values relative to *β-ACTIN* in Figure 4a.**

| \| Gene \| Condition \| Expression level relative to *β-ACTIN* \| \| --- \| --- \| --- \| \| *MUC2* \| Ctrl-IOM \| 1·13E-05 (7·71E-06-1·3E-05) \| \| PGE_2_-IOM \| 2·7E-05 (1·7E-05-7·5E-05) \| \| Ctrl-dm \| 1·04E-04 (8·25E-05-1·1E-04) \| \| PGE_2_-dm \| 5·61E-04 (4·57E-04-7·32E-04) \| \| *MUC5B* \| Ctrl-IOM \| 9·53E-06 (7·0E-06-1·2E-05) \| \| PGE_2_-IOM \| 7·2E-05 (4·8E-05-1·2E-04) \| \| Ctrl-dm \| 8·05E-05 (6·47E-05-1·56E-04) \| \| PGE_2_-dm \| 8·48E-04 (6·62E-04-1·38E-03) \| \| *MUC5AC* \| Ctrl-IOM \| 6·88E-05 (6·47E-05-9·61E-05) \| \| PGE_2_-IOM \| 7·82E-04 (6·01E-04-1·05E-03) \| \| Ctrl-dm \| 4·82E-05 (2·94E-05-5·68E-05) \| \| PGE_2_-dm \| 1·68E-03 1·37E-03-2·25E-03) \| \| *CA II* \| Ctrl-IOM \| 0·039 (0·03-0·05) \| \| PGE_2_-IOM \| 0·053 (0·04-0·07) \| \| Ctrl-dm \| 2·79 (1·84-4·62) \| \| PGE_2_-dm \| 3·77 2·6-4·54) \| \| *CHGA* \| Ctrl-IOM \| N.D. (Ct value: 40) \| \| PGE_2_-IOM \| \| Ctrl-dm \| 7·09E-04 (3·27E-04-8·68E-04) \| \| PGE_2_-dm \| 1·48E-04 (3·09E-05-2·19E-04) \| |
| --- | --- | --- | --- | --- | --- | --- | --- | --- | --- | --- | --- | --- | --- | --- | --- | --- | --- | --- | --- | --- | --- | --- | --- | --- | --- | --- | --- | --- | --- | --- | --- | --- | --- | --- | --- | --- | --- | --- | --- | --- | --- | --- | --- | --- | --- | --- | --- |

Gene expression of mucins (*MUC2*, *MUC5B*, and *MUC5AC,* which are markers for goblet cells), *CAII* (enterocyte marker), and *CHGA* (enteroendocrine marker) were measured by qPCR. N.D. = not detected.
